# Boosting the Capacitance of Covalent Organic Framework Supercapacitors by Hydroquinone Redox Electrolyte Addition

**DOI:** 10.3390/gels10110705

**Published:** 2024-10-31

**Authors:** Laura Sierra, Jesús Á. Martín-Illán, Félix Zamora, Pilar Ocón

**Affiliations:** 1 Departamento de Química-Física Aplicada, Universidad Autónoma de Madrid, 28049 Madrid, Spain; pilar.ocon@uam.es; 2 Departamento de Química Inorgánica, Universidad Autónoma de Madrid, 28049 Madrid, Spain; j.a.martinillan@gmail.com; 3 Institute of Condensed Physic Matter (IFIMAC), Universidad Autónoma de Madrid, 28049 Madrid, Spain

**Keywords:** hydroquinone, pseudocapacitor, aerogel

## Abstract

Rapidly escalating energy demands have spurred a relentless quest for innovative materials and methodologies in energy storage technologies. Covalent organic frameworks (COFs) have emerged as promising candidates for energy storage applications owing to their customizable structure and inherent properties, including enduring porosity and expansive surface area. In this study, we introduce imine-based COF aerogels fashioned into flexible COF electrodes, employing redox electrolytes based on hydroquinone (HQ) dissolved in H_2_SO_4_ aqueous solution and 0.25 M TBAPF_6_ at concentration in acetonitrile. This strategic selection of electrolytes aims to augment capacitance and energy density when compared to non-redox electrolytes. Remarkably, our COF electrodes exhibit an outstanding areal capacitance of 843 mF cm^−2^ when utilizing HQ with 0.10 M H_2_SO_4_, operating at 1.3 mA cm^−2^, while maintaining approximately 100% capacity retention after 10,000 cycles. Notably, the capacitance of the 0.38 M HQ + 0.10 M H_2_SO_4_ is eight times greater than that achieved with organic electrolytes (111 mF cm^−2^).

## 1. Introduction

In the realm of energy storage, the quest for efficient and sustainable solutions has led to the emergence of novel technologies, particularly in the form of batteries and supercapacitors. Positioned as the proverbial bridge between non-renewable and renewable energy sources, these emerging energy storage systems hold tremendous promise for reshaping our energy landscape. Among these, supercapacitors (SCs) stand out for their exceptional attributes: high-power density, rapid charge–discharge capabilities, prolonged lifecycle, and versatility across a wide range of operating temperatures [1].

Supercapacitors come in three primary forms: electrochemical double-layer capacitors (EDLCs), pseudocapacitors (PCs), and hybrid capacitors (HCs). While EDLCs rely on reversible ion adsorption/desorption processes for energy storage, PCs leverage surface-controlled faradaic reactions of redox-active materials at the electrode–electrolyte interface [2]. Both configurations offer distinct advantages, with EDLCs typically employing high-surface-area porous carbons, like activated carbons, excelling in power density and cycle life, while PCs exhibit higher capacitance and energy density, albeit at the expense of power [3,4]. Finally, hybrid capacitors increase capacitance using one battery like an electrode and one electrode like a double-layer capacitor based on carbon given the rise in high capacitance values [5].

However, traditional supercapacitors face limitations, prompting exploration of alternative materials and configurations. Covalent organic frameworks (COFs), dynamic reticular structures formed by organic molecules linked via covalent bonds, have emerged as promising candidates. Possessing permanent porosity and crystallinity, COFs offer tailored porosity, large surface areas, and structural diversity, making them attractive for energy storage applications [6]. Yet, their limited electrical conductivity necessitates enhancements for practical implementation.

To address this, researchers have explored various strategies, including incorporating conductive additives like carbon super P, graphene [7], or conductive polymers [8] into COFs. However, most COFs have been studied just as pseudocapacitor electrodes [9,10]. Moreover, the introduction of redox-active compounds into electrolytes has shown significant potential for enhancing supercapacitor performance. By enabling redox reactions within the electrolyte, these additives complement the charge storage mechanisms within the electrode, leading to synergistic improvements in capacitance and energy density [11,12].

Recent studies have underscored the effectiveness of redox electrolytes in conjunction with porous electrodes [13,14,15], facilitating the diffusion of redox species throughout the electrode and enhancing overall capacitance [16]. The appearance of redox-additive material in the aqueous electrolyte for electrochemical capacitors was first reported by Thanashi et al. [17]. Both inorganic and organic compounds have been explored as redox species, offering a spectrum of options tailored to specific solvent and electrode requirements. The inorganic redox compounds mainly used include halogen ions (e.g., iodide, bromide), metal ions such as Cu^2+^ and Fe^2+^ [18], and metal complexes Fe(CN)_6_ ^3−^ and Fe(CN)_6_ ^4−^ [19,20]. Kushwaha et al. in 2021 presented for the first time an improvement in the capacitance in COFs based on redox electrolytes using KI and 1 M H_2_SO_4_ as redox electrolytes, with an increase in capacitance from 41 mF cm^−2^ for PVA-H_2_SO_4_ gel electrolyte to 270 mF cm^−2^ for PVA-H_2_SO_4_ and KI [21]. On the other hand, organic compounds used as redox electrolytes stand out mainly as methylene blue, catechol, benzoquinone, and hydroquinone [22,23,24]. HQ is an excellent additive with the potential to enhance the specific capacitance substantially. The hydroxyl functional group in HQ exhibits an affinity for the functional groups on the surface of COFs. This interaction can foster a diverse array of hydrogen bonds with the amino groups within the COF structure, provided that the COF contains these amino groups. Roldán et al. demonstrated a significant increase in the specific capacitance of EDLC based on active carbon from 320 F g^−1^ with H_2_SO_4_ to 901 F g^−1^ using hydroquinone HQ and H_2_SO_4_ redox electrolyte [13]. Conversely, dual redox electrolytes have been used to enhance capacitance and energy [25]. For instance, Yong Chen et al. employed electrolytes based on Na_2_MoO_4_ and KBr to achieve good capacitance and energy while maintaining Coulombic efficiency [26].

Herein, we present an imine-based COF aerogel (AGCOF) supercapacitor based on 2,4,6-tris(4-aminophenyl)-1,3,5-triazine (TZ), 1,3,5-benzenetricarbaldehyde (BTCA) mixed with carbon super P, and COF electrodes (ECOFs) TZ-BTCA-AGCOF, augmented by HQ as an electrochemical redox-active mediator in both acidic and organic electrolytes. Through a comprehensive electrochemical characterization employing techniques such as cyclic voltammetry, galvanostatic charge–discharge, and electrochemical impedance spectroscopy, we unveil the exceptional capacitance of 843 mF cm^−2^ achieved in HQ and 0.10 M H_2_SO_4_ at 1.3 mA cm^−2^. This work highlights the potential of COF aerogels with redox electrolytes in advancing supercapacitor technology, offering insights into the design of high-performance energy storage systems crucial for the transition towards a sustainable energy future.

## 2. Results and Discussion

The CV test was performed in a symmetric supercapacitor for each electrolyte to choose the correct potential window and analyze the pseudocapacitive behavior due to the addition of HQ. Furthermore, we evaluated CVs at low scan rates for both electrolytes to analyze the reversibility of the redox process and the possible interaction between the electrode and the HQ.

**Figure 1:**
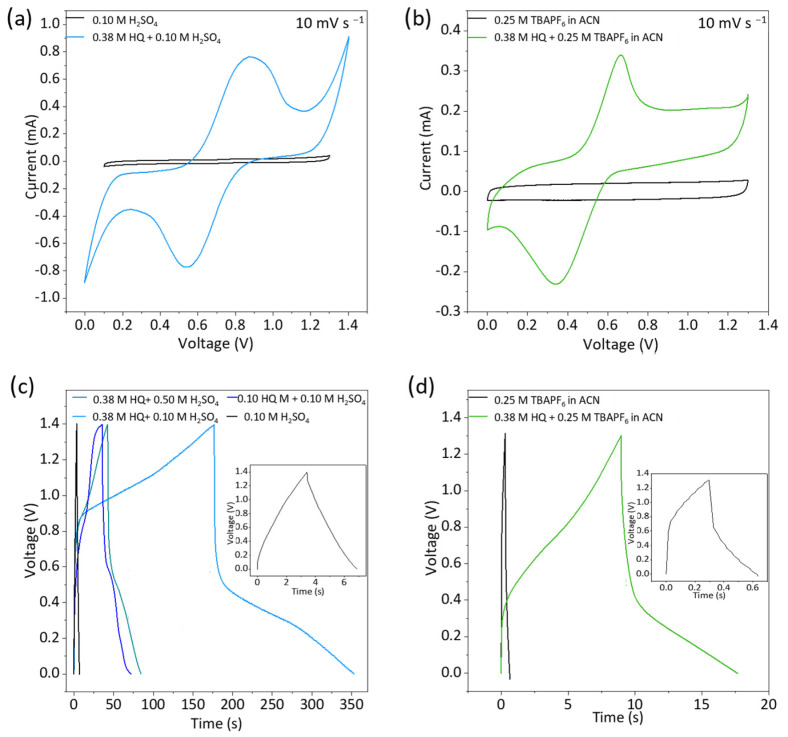
Cyclic voltammetry of (**a**) 0.10 M H_2_SO_4_ (black), 0.38 M HQ + 0.10 M H_2_SO_4_, and 0.10 M HQ (blue) (**b**) 0.25 M TBAPF_6_ in ACN (black) and 0.38 M HQ + 0.25 M TBAPF_6_ in ACN (green). Galvanostatic charge–discharge at 2.0 mA cm^−2^ cycle 10,000. (**c**) 0.10 M H_2_SO_4_ (black), 0.50 M HQ + 0.10 M H_2_SO_4_ (cyan), 0.38 M HQ + 0.10 M H_2_SO_4_ (light blue), 0.38 M HQ + 0.10 M H_2_SO_4_ (dark blue), (**d**) 0.25 M TBAPF_6_ in ACN (black), and 0.38 M HQ + 0.25 M TBAPF_6_ in ACN (green). Inset (**c**) enlargement of 0.10 M H_2_SO_4_ (black). Inset (**d**) enlargement of 0.25 M TBAPF_6_ in ACN (black).

**Scheme 1:**
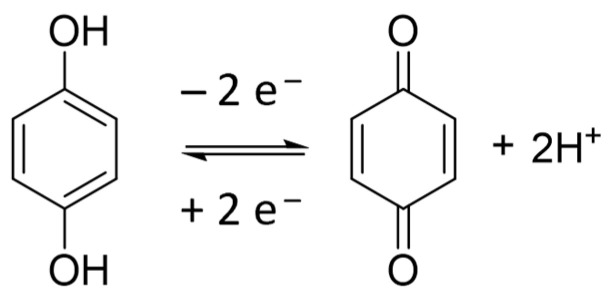
Hydroquinone (HQ) quinone (Q) redox reaction.

Figure 1a,b show the influence of the HQ addition in both electrolytes. The CV at 10 mV s^−1^ shows a remarkable change, and an extra-large area under the voltammogram with HQ appears compared to neat electrolytes. There is an ideal rectangular shape in 0.10 M H_2_SO_4_ and 0.25 M TBAPF_6_ in ACN with no visible redox peak characteristic of a perfect EDLC over the whole potential region studied [27] until the appearance of broad and symmetric redox peaks (centered at E_1/2_ = 0.65 V (aqueous media) and E_1/2_ = 0.53 V (organic media)) correspond to the redox process (Scheme 1). This reaction occurs mainly in the anode side as previously discussed in reference [13].

Based on an examination of the potential profiles in the charge–discharge process at 2 mA cm^−2^, the Figure 1c,d inset shows the curves for pure EDCL (without HQ addition) characteristic of a symmetric supercapacitor, with one single storage mechanism in both electrodes having the same charge storage capacity approximately. However, with the addition of HQ, the characteristic plateaus of the redox reaction at a constant potential appear with a longer charge–discharge time than in the former (Figure 1c,d). This is based on the formal potential of the HQ according to a faradaic mechanism. These results are in addition to the double-layer capacitance and Faradic pseudo-capacitance. The HQ-COF materials show longer discharge times in both electrolytes, which means that they possess higher specific capacitance. At the beginning of discharge, there are a few sudden potential drops (IR drop), as the results of many researchers [28,29,30] are attributed to the ohmic resistance of electrolytes and inner resistance of ion diffusion in pores.

To choose the best electrolyte composition in an aqueous medium, three different essays were analyzed using the performance of GCD at a constant current of 2.0 mA cm^−2^. We used 0.5 M H_2_SO_4_ + 0.38 M HQ, 0.10 M H_2_SO_4_ + 0.10 M HQ, and 0.10 M H_2_SO_4_ + 0.38 M HQ to obtain the areal capacitance values of 103 mF cm^−2^, 89 mF cm^−2^, and 415 mF cm^−2^, respectively, and the last one presented the best option (see Figure 1c). According to the literature, HQ works well from a kinetic point of view in acid pH, even in 1 M of H_2_SO_4_ [14,31]. On the other hand, increasing the concentration of H_2_SO_4_ increases the amount of ionic charge carriers, H^+^, thus improving the EDCL capacity of the COF. However, in this case, 0.50 M H_2_SO_4_ + 0.38 M HQ becomes worse. This effect may be related to the chemical/mechanical stability of the TZ-BTCA-ECOF since, as was indicated in our previous work [27], the stability in 1 M H_2_SO_4_ was compromised. Regarding the increase in the HQ concentration, the maximum solubility found in both media was 0.38 M, so this composition was not exceeded.

Figure S1 shows the GCD corresponding to 0.10 M H_2_SO_4_ and 0.25 M TBAPF_6_ without HQ at different cycles showing high-capacity retention and stability in both electrolytes. The capacitance values were enhanced from 8.5 mF cm^−2^ for 0.10 M H_2_SO_4_ to 415 mF cm^−2^ for 0.38 M HQ + 0.10 M H_2_SO_4_ at 2.0 mA cm^−2^ in the cycle 10,000, and from 1.5 mF cm^−2^ for 0.25 M TBAPF_6_ in ACN to 22 mF cm^−2^ for 0.38 M HQ and 0.25 M TBAPF_6_ at the same conditions.

**Figure 2:**
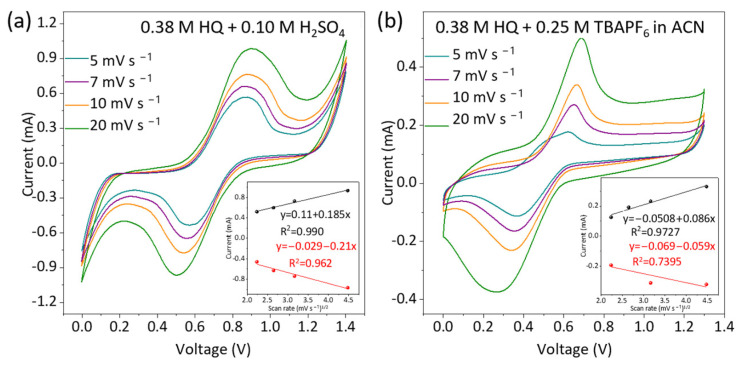
Cyclic voltammetry at different scan rates of (**a**) 0.38 M HQ +0.10 M H_2_SO_4_ and (**b**) 0.38 M HQ + 0.25 M TBAPF_6_ ACN. Inset in both (**a**,**b**) current vs. v^−1/2^.

CVs at different scan rates (5–20 mV s^−1^) were carried out to analyze the kinetics of electrode reactions in the presence of HQ. Figure 2a,b, for 0.38 M HQ+ 0.10 M H_2_SO_4_, show peak–peak potential separation, ΔE_p_ (E_anodic peak_ − E_cathodic peak_), where the values are 0.26–0.39 V for 5–20 mV s^−1^ and 0.25–0.43 V for HQ 0.38 M + TBAPF_6_ 0.25 M in can, respectively. These values increase gradually when the scan rate goes up, possibly due to the increase in electrochemical polarization. The kinetic analysis of the electrode, in the presence of HQ, was evaluated through the Randles–Sevcik plot (i_p_ vs. v^1/2^) for both media [32]. In the case of 0.38 M HQ and 0.10 M H_2_SO_4_ (Figure 2a), a linear trend indicates that the HQ charge–transfer process seems to be diffusion-controlled. In contrast, for 0.38 M HQ in 0.25 M TBAPF_6_ (Figure 2b), the plot (i_p_ vs. v^1/2^) does not follow a linear trend, indicating that some surface reaction could control the redox process [33].

The capacitance values obtained from CV at different scan rates (5–100 mV s^−1^) are included in Figures S3 and S4. For 0.38 M HQ + 0.10 M H_2_SO_4_, the capacitance significantly decreases when the scan rate increases, achieving the best value of 121 mF cm^−2^ at 5 mV s^−1^; however, at 100 mV s^−1^, the value is 31 mF cm^−2^. For 0.38 M HQ in 0.25 M TBAPF_6_, the higher value was obtained at 7 mV s^−1^ (35 mF cm^−2^) and then decreased to 20 mF cm^−2^ at 100 mV s^−1^. Nevertheless, the capacitance variation with the scan rates is less significant than in acid media, probably because the kinetics reaction is different in both cases.

For the organic electrolyte, the capacitance from GCD at 2.0 mA cm^−2^ also increases from 1.3 mF cm^−2^ to 36 mF cm^−2^ for 0.25 M TBAPF_6_ in ACN electrolyte and 0.38 M HQ + 0.25 M TBAPF_6_, respectively.

**Figure 3:**
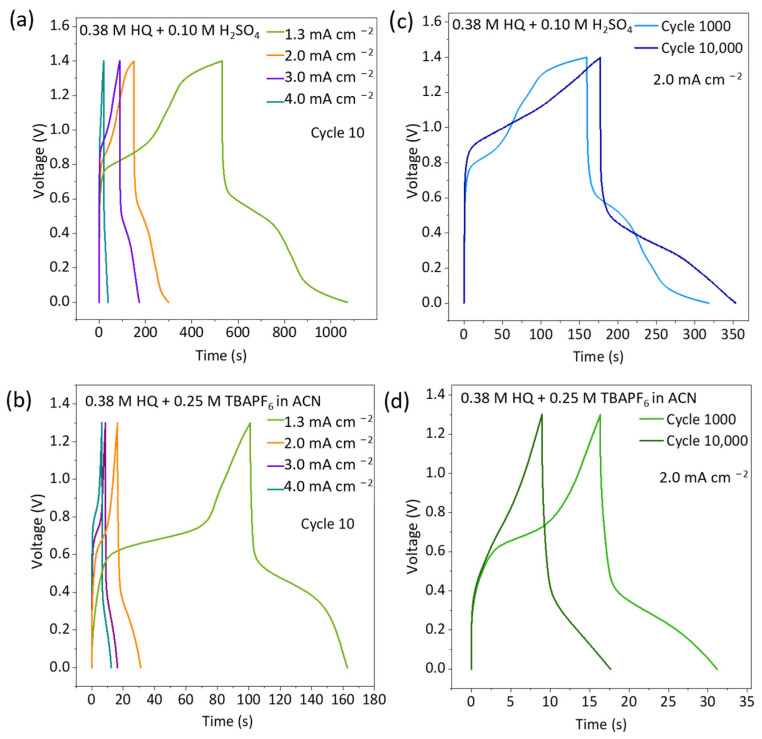
Galvanostatic charge–discharge (**a**) 0.38 M HQ + 0.10 M H_2_SO_4_ at different rates, (**b**) 0.38 M HQ + 0.25 M TBAPF_6_ in ACN at different rates, (**c**) 0.38 M HQ + 0.10 M H_2_SO_4_ at constant current 2.0 mA cm^−2^, and (**d**) 0.38 M HQ + 0.25 M TBAPF_6_ in ACN at constant current 2.0 mA cm^−2^.

Furthermore, the behavior based on the GCD test was analyzed for both electrolytes at different current rates (1.3, 2.0, 3.0, 4.0 mA cm^−2^) for acid aqueous and organic electrolyte, shown in Figure 3a,b, respectively. In both cases, the capacitance values decrease when the current rate increases (Table S1 and Figure S5), showing the best values of 843 mF cm^−2^ at 1.3 mA cm^−2^ and 111 mF cm^−2^ at 4.0 mA cm^−2^ for aqueous and organic electrolyte, respectively. Additionally, the CE% was evaluated for both electrolytes at different current rates. For 0.38 M HQ + 0.10 M H_2_SO_4_, the CE% is 98% at the lowest current (1.3 mA cm^−2^) and 99% at the higher currents; this indicates the good efficiency of the redox process. On the other hand, for 0.38 M HQ + 0.25 M TBAPF_6_ in can, the CE% is poor, 61% for (1.3 mA cm^−2^) and 99% at higher currents, indicating the lower efficiency of the redox process.

Figure 3c shows the corresponding GCD curves for 0.38 M HQ + 0.10 M H_2_SO_4_ for the whole cell. The comparison between cycle 1000 and cycle 10,000 shows a capacity retention of around 100%; the capacitance increases slightly, showing a final value of 415 mF cm^−2^. Moreover, the GCD curves for 0.38 M HQ + 0.25 M TBAPF_6_ in ACN show a significant loss after 10,000 cycles and a capacitance retention of almost 54.5%. The GCD and CE% results at a constant current rate and different cycles are recorded in Table S2. At 2.0 mA cm^−2^, the CE% reaches 99% from cycle 1000 for 0.38 M HQ + 0.10 M H_2_SO_4_; however, for the case of 0.38 M HQ + 0.25 M TBAPF_6_, this value is reached after 3000 cycles.

The values obtained by GCD in the 0.38 M HQ + 0.10 M H_2_SO_4_ electrolyte at 1.3 mA cm^−2^ are significantly higher than previously obtained with other COF structures that incorporate the same hydroquinone functional group in the structure; these values are shown in Table 1.

Additionally, to demonstrate the contribution of the COF material to the excellent performance of the device, a cell in symmetric configuration with carbon super P electrodes was performed (Figure S2). In this case, the total amount of active material carbon super P was 5 mg (77 wt %) and PVDF 1.5 mg (33 wt %). The capacitance obtained was 94.6 mF cm^−2^ at a current density of 2.0 mA cm^−2^ at the beginning of the cycling, but in cycle 1500, this value decreased to 49.8 mF cm^−2^ and lost the redox shape of the HQ. This demonstrates the beneficial effect of COF aerogel on the capacity results, considering that the amount of carbon in TZ-BTCA-ECOF is 30%.

**Figure 4:**
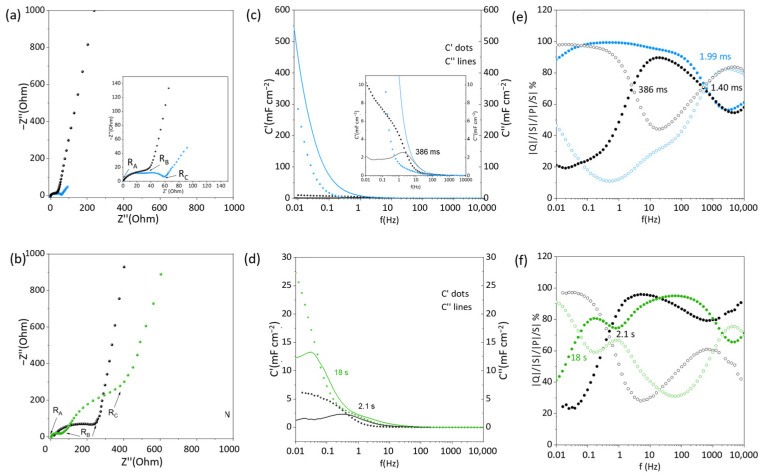
Top EIS after 1000 cycles for 0.10 M H_2_SO_4_ (black) and 0.38 M HQ + 0.10 M H_2_SO_4_ (blue) and down 0.25 M TBAPF_6_ in ACN (black) and 0.38 M HQ + 0.25 M TBAPF_6_ in ACN (green). (**a**,**b**) Nyquist plot. (**c**,**d**) C′ and C″ vs. frequency plot and (**e**,**f**) active and reactive power (P/S), (Q/S).

**Figure 5:**
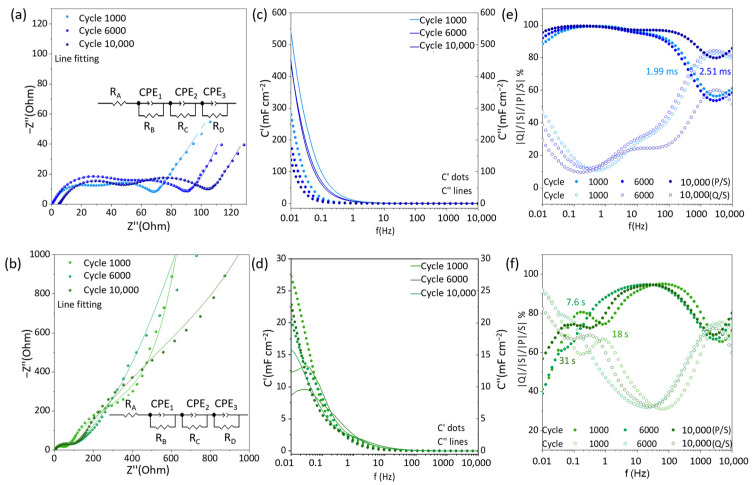
Top EIS 0.38 M HQ + 0.10 M H_2_SO_4_ and down 0.38 M HQ + 0.25 M TBAPF_6_. (**a**,**b**) Nyquist plot, (**c**,**d**) C′ and C″ vs. frequency plots, and (**e**,**f**) active and reactive power (P/S), (Q/S).

Furthermore, the changes during the GCD process at a constant current rate were analyzed through EIS for both electrolytes. The Nyquist plot gives information about the phenomena that occur during the charge–discharge process concerning the resistances. In the case of the materials without HQ, meaning without faradaic reactions, R_A_ (located at a high frequency) represents the intercept on the real axis and provides information about the contribution of the resistances originating from the interaction between the electrode and electrolyte and the contact resistance between the electrode material and the current collector; R_B_ (located in the middle frequency, approximately at 20 Hz) is associated with the porous structures of COF materials. On the other hand, the ion transport limitation could be found in the middle–low frequency, and finally, the capacitive behavior appears [34]. For the electrolyte with HQ, the Nyquist plot changes, and a new semicircle between 40 and 0.2 Hz appears, which reflects the HQ redox process, resulting in the diffusional and/or capacitive part at lower frequencies than without HQ. Following previous examples of these kinds of materials^36^, the corresponding equivalent series circuits (included in Figure 4 and Figure 5) have been checked; R_A_(CPE_1_/R_B_)(CPE_3_/R_D_) for electrolyte without HQ and R_A_(CPE_1_/R_B_) (CPE_2_/R_C_) (CPE_3_/R_D_) electrolyte with HQ. The first R/C represents the porous structure, the second R/C represents the HQ redox reaction, and the third R/C represents the system’s diffusional and/or capacitive part. The analysis allows for the identification of different EIS parameters for interpretation, and the corresponding fitting parameters are collected in Table S3.

First of all, the EIS measurements after 1000 cycles during the constant GCD at 2.0 mA cm^−2^ for electrolytes with and without HQ are compared (Figure 4a,b). The Nyquist plot of 0.10 M H_2_SO_4_ without and with 0.38 M HQ show the same R_A_ values 0.38 Ω; meanwhile, in (R_B_/CPE_1_), semicircle R_B_ is higher for 0.10 M H_2_SO_4_, 36 Ω vs. 28 Ω, and the second semicircle (R_C_/CPE_2_) which is observed when the HQ is added is not present. Finally, low-frequency 0.10 M H_2_SO_4_ presents a vertical line attributed to the ideal capacitive (n_3_ = 0.86); however, with HQ, this aspect is found at lower frequencies (0.2 Hz), and the ideal capacitor behavior is lost (n_3_ = 0.75).

The Nyquist plot of 0.25 M TBAPF_6_ in ACN and 0.38 M HQ + 0.25 M TBAPF_6_ can show R_A_ values of 12.0 Ω and 3.6 Ω, respectively. A higher R_B_ resistance of 257 Ω (5 Hz) for the organic electrolyte without HQ vs. 49 (40 Hz) with HQ was observed. As we discussed in our previous work [27], no diffusional parts are associated with the porous material of the organic electrolyte. This is because the organic salts present ions with an ion size larger than the intrinsic pore size, so the capacitance is lower in the organic electrolyte [26]. Moreover, like H_2_SO_4_ 0.10 M without HQ, a vertical line attributed to ideal capacitive behavior (n_3_ = 0.90) is found at a low frequency, with a small frequency delay with the addition of HQ being reported on the ideal capacitive (n_3_ = 1.00) performance. Figure S6 shows the equivalent circuit for all the electrolytes.

On the other hand, EIS measurements are recorded at 6000 and 10,000 cycles to understand the evolution with cycling time (Figure 5a,b). For 0.38 M HQ + 0.10 M H_2_SO_4_, R_A_ increases to 5.6 Ω after 10,000 cycles. That increase in resistance can be attributed to changes in the electrode during the charge–discharge process. For the organic electrolyte with HQ, R_A_ values are higher than for the acid electrolyte due to the lower conductivity of the organic electrolyte, but finally, it obtained the same R_A_ 5.57 Ω after 10,000 cycles. Next, R_B_ increases from 30 Ω (50 Hz) to 46 Ω (63 Hz) after 10,000 cycles, and R_C_ increases from 68 Ω (0.39 Hz) to 105 Ω (0.19 Hz). As can be observed in the GCD (Figure 3c), there was a faradaic loss contribution, and consequently, the system became more resistive. On the other hand, R_B_ in organic electrolyte with HQ changes from 49 (100 Hz) to 83 Ω (31 Hz), and R_C_ increases from 557 Ω (0.15 Hz) to 1837 Ω (0.05 Hz). The changes observed are related to the significant loss of capacitance in the 10,000 cycles, which are always larger than in aqueous media. It should be noted that at low frequencies, for HQ 0.38 M + H_2_SO_4_ 0.10 M, CPE_3_ n_3_ decreases to 0.68.

The complex capacitance allows for a better perspective study of the capacitance changes with the frequencies. The real part (C′) gives information about the changes in the capacitance; meanwhile, the imaginary part (C″) is used to extract information about the energy dissipation in the systems [35]. Figure 4c shows C′ and C″ vs. frequency; at 0.01 Hz, the maximum capacitance value is 286 mF cm^−2^ for 0.38 M HQ + 0.10 M H_2_SO_4_, while for H_2_SO_4_, this value is 10 mF cm^−2^. Also, a maximum is attributed to τ_0_ for 0.10 M H_2_SO_4_ at 386 ms. With HQ, the maximum is shifted to lower frequencies, as previously mentioned, and it is not detectable at the working frequency limits (Figure 5c). For organic electrolytes at the frequency 0.01 Hz, the maximum capacitance with HQ is 275.0 mF cm^−2^ and 5.0 mF cm^−2^ without HQ, increasing the τ_0_ value from 2.1 s to 18.0 s with the addition of HQ. This rising value of τ0 is associated with the presence of faradic reactions because this process needs more time to take place on the surface of the electrode.

From Figure 5c,d, the decrease in C’s share from 286 mF cm^−2^ in cycle 1000 to 137 mF cm^−2^ for cycle 10,000 is associated with increased resistance in the system. For 0.38 M HQ + 0.25 M TBAPF_6_, C′ varies from 30 to 22 mF cm^−2^; those changes can be due to the degradation of the redox electrolyte during the charge–discharge process. On the other hand, for C″, there is not a maximum corresponding to τ_0_ for 0.38 M HQ + 0.10 M H_2_SO_4_ in the low frequencies, and in 0.38 M HQ + 0.25 M TBAPF_6_, the maximum appears at 31 s after 10,000 cycles.

Finally, the power dissipated into the system can be analyzed by the normalized real part of the complex power, active power (|P|/|S|), and normalized imaginary part reactive power (|Q|/|S|). The (|P|/|S|) corresponded to the power dissipated into the system. When all the power is dissipated (P 100%), the supercapacitor or the EDLC behaves like pure resistance; this occurs at high frequencies (Figure 4e,f). The (|Q|/|S|) increases when the frequency decreases, and pure capacitance exhibits only reactive power Q (Equations (S6) and (S7)). The maximum of (|Q|/|S|) is reached at low frequency when the ECDL behaves like a pure capacitor. Figure 4e,f show two crossing points for 0.10 M H_2_SO_4_, at a high frequency, 1.40 ms, like the system with HQ, and another in the middle frequency at 386 ms, but with HQ, the crossing point is outside the working frequency. Finally, τ0 shifts to 2.1 s from 18 s with the addition of HQ for the organic electrolyte. The time constant τ_0_ marks the boundary between resistive and capacitive behavior. The HQ addition in both electrolytes moves the time constant to higher values, causing the energy stored in the device to be delivered more slowly, as is otherwise more typical of the pseudocapacitive behavior provided by the redox couple mediator.

Finally, Figure 5e,f represent the normalized power vs. frequency for different numbers of cycles [36]. For 0.38 M HQ + 0.25 M TBAPF_6_ at a high frequency, all reactive power is dissipated, while for 0.38 M HQ + 0.10 M H_2_SO_4_, this does not happen (Figure 5e,f); this may be because it presents a battery-like behavior instead of pure capacitive. As we previously described, the crossing points moves to higher values of τ_0_, outside of the range of frequency that it was measured in, so the main change showing after 10,000 cycles is that the first crossing point observed around τ_0_ = 2 ms disappears, losing all the character corresponding to a pure EDLC [37]. In contrast, for 0.38 M HQ + 0.25 M TBAPF_6_, the crossing point occurs at low frequencies varying from 18.0 s after 1000 cycles to 31.0 s after 10,000 cycles.

The electrochemical study of the SCs with the addition of HQ in the different electrolytes has shown that the synergic effect between the use of COF electrodes and HQ as a redox electrolyte significantly improves the total capacitance of the devices as well as the energy density, which gives rise to an alternative application of this material in the field of energy storage devices.

## 3. Conclusions

In this study, we have showcased a significant enhancement of capacitance and energy storage capacity through the strategic substitution of inert electrolytes with appropriate redox electrolytes, thereby harnessing the synergy between electrochemical double-layer capacitance and Faraday reactions. Specifically, our investigation focused on the integration of TZ-BTCA-ECOF electrodes with an electrolyte comprising 0.38 M HQ + 0.10 M H_2_SO_4_, resulting in remarkable capacitance and energy values of 843 mF cm^−2^ and 23.10 mWh cm^−3^, respectively, alongside excellent capacitance retention characteristics.

Furthermore, our EIS analyses provided valuable insights into the dynamic changes occurring within the system upon HQ addition. Notably, the observed increase in resistances at middle–low frequencies (RC) reflects the active participation of redox reactions, while the higher values of τ0 signify predominant pseudocapacitive behavior. Additionally, the EIS measurements conducted during cycling at constant current unveiled instances of electrolyte degradation, highlighting the need for further investigations into electrolyte/electrode stability and long-term performance.

The results presented in this work show high specific capacitance values in comparison with similar HQ groups in other COF structures [9,10,11,12] together with a high capacitance retention.

Overall, our findings underscore the potential of redox electrolytes to significantly enhance the electrochemical performance of supercapacitors, offering a pathway towards the development of robust and efficient energy storage solutions crucial for advancing various technological applications.

## 4. Materials and Methods

Following our previously reported method [26], TZ-BTCA-AGCOF was synthesized with 50 mg of 2,4,6-tris(4-aminophenyl)-1,3,5-triazine (TZ) dissolved in 0.3 mL of dimethylsulfoxide (DMSO) and 6 mL of AcOH with 10% (*v*/*v*) of MiliQ-water. Next, 50 mg of 1,3,5 benzenetricalbadehyde (BTCA) was dissolved in 6 mL of AcOH with 10% (*v*/*v*) of MiliQ-water. The final product was a gel that reacted at 25 °C for 3 days, after which the solvent was exchanged with tetrahydrofuran (THF) and ethanol (EtOH). Finally, a critical point dryer of CO_2_ was used to prepare TZ-BTCA-AGCOF [38]. The symmetrical devices were also made to follow the same procedure we previously described [39]. First, the COF aerogels were gently broken into small pieces and mixed with 30% wt. of carbon super P (carbon-C65) in an agate mortar to enhance the electrical conductivity; the mixture was pressed under 120 MPa for 5 min, giving rise to the flexible free-standing electrode of the corresponding TZ-BTCA-AGCOF, named TZ-BTCA-ECOF [27].

The electrochemical experiments in two symmetrical electrodes were performed in a Swagelock-type cell for acid electrolyte and Coin-Cell 2032 for organic electrolyte built-in a glove box with two identical electrodes (3.5 mg of active material on 0.50 cm^2^), Whatman GF/C as the separator, and the corresponding electrolyte. The behavior of TZ-BTCA-ECOF electrodes was analyzed with two different media: acid electrolyte (0.10 M H_2_SO_4_) and organic electrolyte acetonitrile (ACN) with 0.25 M tetrabutylammonium hexafluorophosphate (TBAPF_6_). In the aqueous media, different concentrations of HQ and acid were tested: (i) 0.38 M HQ + 0.5 M, H_2_SO_4_ (ii) 0.10 M HQ and 0.10 MH_2_SO_4_, and (iii) 0.38 M HQ and 0.10 M H_2_SO_4_. Also, we tested 0.38 M HQ and 0.25 M TBAPF_6_ for organic media in ACN.

CV, GCD, and EIS were performed using Biologic multichannel potentiostatic–galvanostatic. CV was carried out in the potential window (0 to 1.4 V) for HQ + H_2_SO_4_ and (0 to 1.3 V) for HQ 0.38 M + TBAPF_6_ 0.25 M in ACN at room temperature at different scan rates (5 to 100 mV s^−1^). A galvanostatic charge–discharge test (GCD) was performed between the same potential window and at constant current densities of 1.3, 2.0, 3.0, and 4.0 mA cm^−2^ at room temperature. Capacitance values have been normalized by the geometric area of the COF material, resulting in area capacitance (mF cm^−2^). Electrochemical impedance spectroscopy (EIS) was carried out in the frequency range of 10^4^ to 10^−2^ Hz with an Δrms of 10 mV. All measurements were made at the resting potential of the cell.

## Figures and Tables

**Table 1:** Capacitance, energy, and cycling stability of different COF structures with hydroquinone.

Active Material	Electrolyte	Capacitance (mF cm^−2^)	Energy (µW h cm^−2^)	Cycling Stability	Ref.
Dq_1_Da_1_Tp COF (Anthraquinone)	PVA-H_2_SO_4_ gel electrolyte	8.5 (0.39 mA cm^−2^)	0.3	78% (7000)	[9]
Dq_1_Tp COF (Anthracene)	PVA-H_2_SO_4_ gel electrolyte	12 (0.39 mA cm^−2^)	0.4	80% (2500)	[10]
TpOMe-DAQ COF	PVA-H_2_SO_4_ gel electrolyte	84 (0.25 mA cm^−2^)	2.9	90% (10,000)	[11]
TpPa-(OH)_2_ COF	1.0 M phosphate buffer	98 (1.3 mA cm^−2^)	19.4	88% (10,000)	[12]
TZ-BTCA-ECOF	0.38 M HQ + 0.10 M H_2_SO_4_	843 (1.3 mA cm^−2^)	231	99% (10,000)	This work
TZ-BTCA-ECOF	0.38 M HQ + 0.25 M TBAPF_6_	111 (1.3 mA cm^−2^)	26.2	61% (10,000)	This work

## Data Availability

The data presented in this study are openly available in article.

## Supplementary Materials

The following supporting information can be downloaded at: https://www.mdpi.com/article/10.3390/gels10110705/s1, Figure S1: GCD of TZ-BTCA-ECOF (a) 0.1 M H_2_SO_4_ and (b) 0.25 M TBAPF_6_ in ACN at 1.3 mA cm^−2^. Figure S2: GCD of Carbon Super P and 0.38 M HQ + 0.1M H_2_SO_4_ at a current density of 2.0 mA cm^−2^. Cycle 100 (black) and cycle 1500 (violet). Figure S3: Areal capacitance (mF cm^−2^) at different scan rates. Figure S4: CV at different scan rates (a) 0.38 M HQ + 0.1 M H_2_SO_4_ and (b) 0.38 M HQ + 0.25 M TBAPF_6_ in ACN. Table S1: Capacitance, and energy parameters at different current rate. Figure S5: Specific capacitance (mF cm^−2^) at different current densities. Table S2: Capacitance, and energy parameters at the constant current rate. Table S3: EIS fit parameters of 0.38 M HQ + 0.1 M H_2_SO_4_ and 0.38 M HQ + 0.25 M TBAPF_6_ Figure S6: Equivalent circuits for (a) 0.38 M HQ + 0.1 M H_2_SO_4_ and 0.38 M HQ 0.25 M 0.25 M TBAPF_6_ (b) 0.1 M H_2_SO_4_ and 0.25 M TBAPF_6_.
