# Supplementary material for: Boosting the Capacitance of Covalent Organic Framework Supercapacitors by Hydroquinone Redox Electrolyte Addition
Source: Gels. 2024 Oct 31;10(11):705. doi: 10.3390/gels10110705 (PMC11594047; doi:10.3390/gels10110705)
Supplement: Supplementary file 1 [file gels-10-00705-s001.zip › gels-3227531-supplementary.pdf]

Boosting the Capacitance of Covalent Organic Framework Supercapacitors by Hydroquinone Redox Electrolyte Addition

Laura Sierra [1,*], Jesús Á. Martín-Illán [2], Félix Zamora [2,3,*] and Pilar Ocón [1]

1 Departamento de Química-Física Aplicada, Universidad Autónoma de Madrid, 28049 Madrid, Spain; pilar.ocon@uam.es
2 Departamento de Química Inorgánica, Universidad Autónoma de Madrid, 28049 Madrid, Spain; j.a.martinillan@gmail.com
3 Institute of Condensed Physic Matter (IFIMAC), Universidad Autónoma de Madrid, 28049 Madrid, Spain
* Correspondence: laura.sierra@uam.es (L.S.) felix.zamora@uam.es (F.Z.)

Supporting information

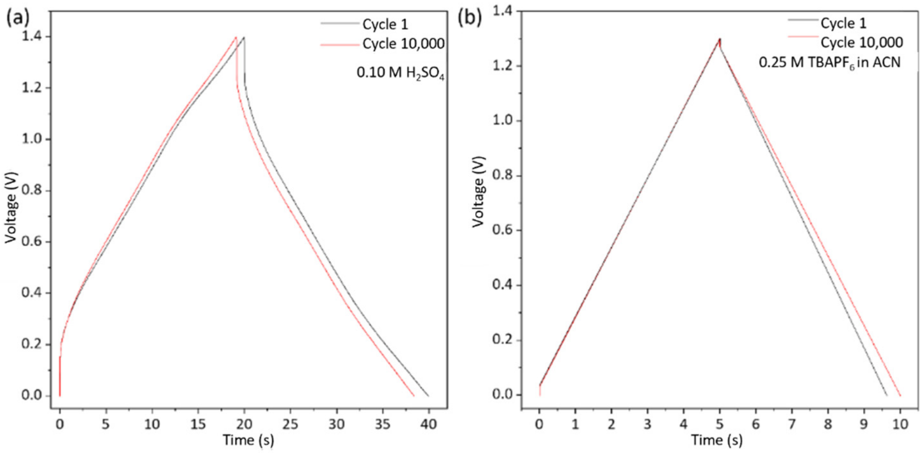

Figure S1. GCD of **TZ-BTCA-ECOF** (a) 0.1 M H_2SO_4 and (b) 0.25 M $TBAPF_6$ in ACN at 1.3 mA cm^{-2}.

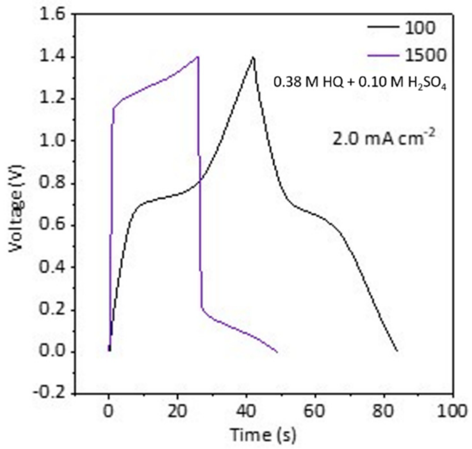

Figure S2. GCD of **Carbon Super P** and 0.38 M HQ + 0.1M H_2SO_4 at a current density of 2.0 mA cm^{-2}. Cycle 100 (black) and cycle 1500 (violet).

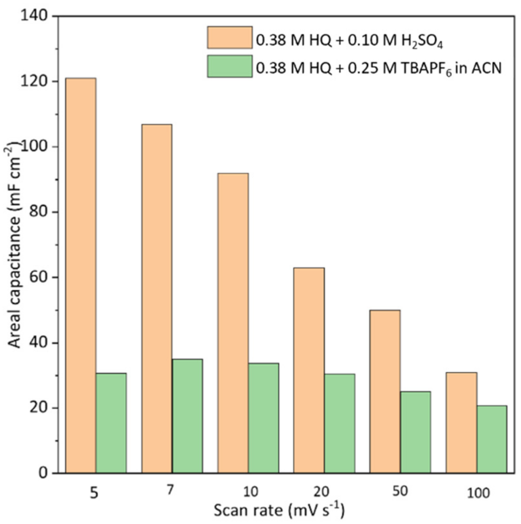

Figure S3. Areal capacitance (mF cm^{-2}) at different scan rates

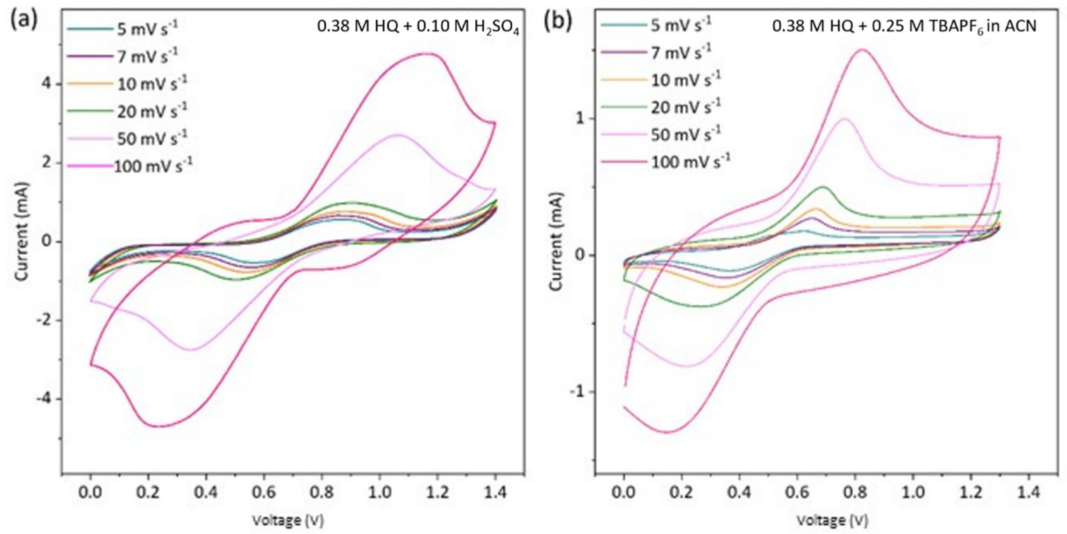

Figure S4. CV at different scan rates (a) 0.38 M HQ + 0.1 M H_2SO_4 and (b) 0.38 M HQ + 0.25 M $TBAPF_6$ in ACN.

Table S1. Capacitance, and energy parameters at different current rate.

0.38 M HQ + 0.1 M H_2SO_4			
**Different current rate**	**Capacitance (mF cm^{-2})**	**Capacitance (F g^{-1})**	**Energy (mWh cm^{-3})**
1.3 mA cm^{-2} (Cycle 10)	843 (98 % CE)	120	23.10
2.0 mA cm^{-2} (Cycle 10)	399 (99 % CE)	55.2	10.91
3.0 mA cm^{-2} (Cycle 10)	382 (99 % CE)	54.9	10.46
4.0 mA cm^{-2} (Cycle 10)	137 (99 % CE)	20.1	3.74
**0.38 M HQ + 0.25 M $TBAPF_6$ in ACN**			
1.3 mA cm^{-2} (Cycle 10)	111 (61%CE)	15.8	2.62
2.0 mA cm^{-2} (Cycle 10)	51 (92% CE)	6.9	1.20
3.0 mA cm^{-2} (Cycle 10)	43 (99 % CE)	5.8	1.01
4.0 mA cm^{-2} (Cycle 10)	40 (99 % CE)	5.4	0.93

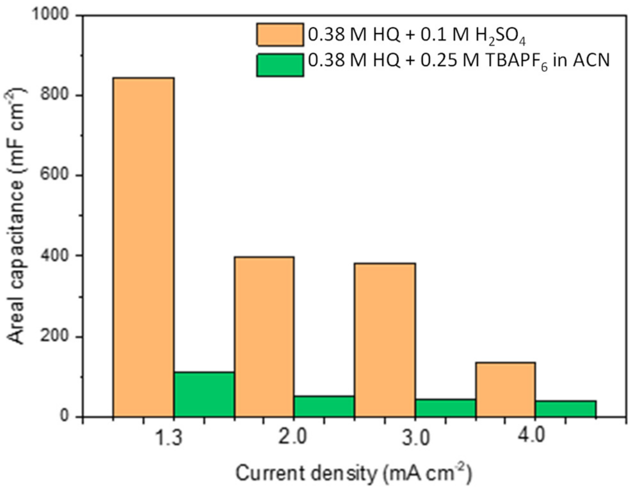

Figure S5. Specific capacitance (mF cm^{-2}) at different current densities.

Table S2. Capacitance, and energy parameters at the constant current rate.

0.38 M HQ + 0.1 M H_2SO_4			
**Constant current rate (2.0 mA cm^{-2})**	Capacitance (mF cm^{-2})	Capacitance (F g^{-1})	Energy (mWh cm^{-3})
Cycle 100	330 (98% CE)	49.2	8.79
Cycle 1000	385 (99% CE)	59.5	10.45
Cycle 3000	382 (99% CE)	58.2	10.39
Cycle 6000	399 (99% CE)	60.7	10.84
Cycle 8000	418 (99% CE)	63.7	11.38
Cycle 10,000	415 (99% CE)	63.2	11.29
**0.38 M HQ + 0.25 M $TBAPF_6$ in ACN**			
Cycle 100	36 (91 % CE)	5.1	0.84
Cycle 1000	40 (92 %CE)	5.7	0.94
Cycle 3000	30 (99 %CE)	4.2	0.70
Cycle 6000	26 (99 %CE)	3.7	0.61
Cycle 8000	26 (99 %CE)	3.6	0.60
Cycle 10,000	22 (99 %CE)	3.1	0.56

The capacitance was calculated by:

$$C = 2 \cdot I_S \frac{\int Vdt}{(\Delta V)2} \quad (Eq.S1) \text{ mF cm}^{-2} \text{ [21]}$$

And energy

$$E = I_S \frac{\int Vdt}{3600\ .L} \quad (Eq.S2) \text{ mWh cm}^{-3} \text{[21]}$$

$$E = I_S \frac{\int Vdt}{3.6} \quad (Eq.S3) \ \mu\text{Wh cm}^{-2}$$

I_s = current density in mA cm^{-2}, $\int Vdt$ = integral area of the discharging part of the GCD curve, ΔV = Potential window (V=Vf -Vi), L = electrode thickness, C = mF cm^{-2}, E = mWh.cm^{-3}. The electrode thickness is 100 µm.

The C‘and C‘‘ parameters were calculated by:

$$C(w) = C'(w) + C''(w) \ (Eq.S3)$$

$$C' = \frac{-Z''(w)}{w|Z(w)^2|} \quad (Eq.S4) \quad C'' = \frac{-Z'(w)}{w|Z(w)^2|} \quad (Eq.S5)$$

C´= real part of complex capacitance, C´´=imaginary part of complex capacitance, Z´= real part of the impedance (Ω), Z´´= imaginary part of the impedance (Ω), ω = angular frequency =2πf, f = frequency in Hz.

And the active power (*P*) and reactive power (*Q*) can be described as:

$$P(w) = wC''(w)|\Delta Vrms|^2 \ (Eq.S6)$$

$$Q(w) = wC'(w)|\Delta Vrms|^2 \ (Eq.S7)$$

Table S3. EIS fit parameters of 0.38 M HQ + 0.1 M H_2SO_4 and 0.38 M HQ + 0.25 M $TBAPF_6$

**0.1 M H_2SO_4**											
	**R_A (Ω)**	**CPE_{-1}**		**R_B (Ω)**	**CPE_{-2}**		**R_C (Ω)**	**CPE_{-3}**		**R_D (Ω)**	**Chi^2**
		T	P (n_1)		T	P (n_2)		T	P (n_3)		
1000	0.38	$2.0\cdot10^{-4}$	0.73	36				$1.8\cdot10^{-3}$	0.86	$1.0\cdot10^{20}$	$2.4\cdot10^{-3}$
**0.38 M HQ + 0.1 M H_2SO_4 0.1 M**											
1000	0.38	$9.4\cdot10^{-5}$	0.75	28	$1.7\cdot10^{-3}$	0.69	41	$1.1\cdot10^{-1}$	0.75	281	$3.4\cdot10^{-5}$
6000	0.33	$4.8\cdot10^{-5}$	0.87	28	$1.8\cdot10^{-3}$	0.52	67	$1.8\cdot10^{-1}$	0.74	$3.4\cdot10^{20}$	$4.8\cdot10^{-5}$
10,000	5.61	$4.3\cdot10^{-5}$	0.75	40	$1.9\cdot10^{-3}$	0.63	60	$1.5\cdot10^{-1}$	0.68	$1.0\cdot10^{20}$	$1.5\cdot10^{-5}$
**$TBAPF_6$ 0.25 M in ACN**											
	**R_A (Ω)**	**CPE_{-1}**		**R_B (Ω)**	**CPE_{-2}**		**R_C (Ω)**	**CPE_{-3}**		**R_D (Ω)**	
1000		T	P (n_1)		T	P (n_2)		T	P (n_3)		
	12	$9.0\cdot10^{-3}$	0.60	257				$1.3\cdot10^{-3}$	0.90	$2.0\cdot10^{17}$	2.9·10-3
**HQ 0.38 M + $TBAPF_6$ 0.25 M in ACN**											
1000	3.85	$1.6\cdot10^{-5}$	0.83	49	$1.6\cdot10^{-3}$	0.64	557	$7.1\cdot10^{-3}$	0.93	$1.2\cdot10^{20}$	$1.1\cdot10^{-3}$
6000	4.51	$3.1\cdot10^{-5}$	0.79	80	$2.7\cdot10^{-3}$	0.49	678	$4.3\cdot10^{-3}$	1.00	4355	$1.5\cdot10^{-3}$
10,000	5.57	$3.2\cdot10^{-5}$	0.77	83	$1.7\cdot10^{-3}$	0.61	1837	$4.3\cdot10^{-3}$	1.00	$1.0\cdot10^{20}$	$8.1\cdot10^{-4}$

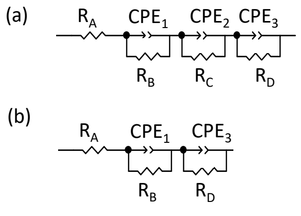

Figure S6. Equivalent circuits for (a) 0.38 M HQ + 0.1 M H_2SO_4 and 0.38 M HQ 0.25 M 0.25 M $TBAPF_6$ (b) 0.1 M H_2SO_4 and 0.25 M $TBAPF_6$
